# Identifying potential drug targets for idiopathic pulmonary fibrosis: a mendelian randomization study based on the druggable genes

**DOI:** 10.1186/s12931-024-02848-5

**Published:** 2024-05-23

**Authors:** Zetao Liu, Zhiyu Peng, Huahang Lin, Ke Zhou, Linchuan Liang, Jie Cao, Zhaokang Huang, Jiandong Mei

**Affiliations:** 1 Department of Thoracic Surgery, West China Hospital, Sichuan University, Chengdu, 610041 China; 2 Western China Collaborative Innovation Center for Early Diagnosis and Multidisciplinary Therapy of Lung Cancer, Sichuan University, Chengdu, China

**Keywords:** Idiopathic pulmonary fibrosis, Mendelian randomization, Druggable genes, *IL-7*

## Abstract

**Background:**

Idiopathic pulmonary fibrosis (IPF) is a chronic fibrotic interstitial lung disease characterized by progressive dyspnea and decreased lung function, yet its exact etiology remains unclear. It is of great significance to discover new drug targets for IPF.

**Methods:**

We obtained the cis-expression quantitative trait locus (cis-eQTL) of druggable genes from eQTLGen Consortium as exposure and the genome wide association study (GWAS) of IPF from the International IPF Genetics Consortium as outcomes to simulate the effects of drugs on IPF by employing mendelian randomization analysis. Then colocalization analysis was performed to calculate the probability of both cis-eQTL of druggable genes and IPF sharing a causal variant. For further validation, we conducted protein quantitative trait locus (pQTL) analysis to reaffirm our findings.

**Results:**

The expression of 45 druggable genes was significantly associated with IPF susceptibility at FDR < 0.05. The expression of 23 and 15 druggable genes was significantly associated with decreased forced vital capacity (FVC) and diffusing capacity of the lungs for carbon monoxide (DLco) in IPF patients, respectively. IPF susceptibility and two significant genes (*IL-7* and *ABCB2*) were likely to share a causal variant. The results of the pQTL analysis demonstrated that high levels of IL-7 in plasma are associated with a reduced risk of IPF (OR = 0.67, 95%CI: 0.47–0.97).

**Conclusion:**

*IL-7* stands out as the most promising potential drug target to mitigate the risk of IPF. Our study not only sheds light on potential drug targets but also provides a direction for future drug development in IPF.

**Supplementary Information:**

The online version contains supplementary material available at 10.1186/s12931-024-02848-5.

## Introduction

Idiopathic pulmonary fibrosis (IPF) is a chronic progressive fibrotic interstitial lung disease with poor prognosis characterized by progressive dyspnea and decline in lung function [1]. In the past two decades, the incidence of IPF has increased, especially among the elderly [2]. Unfortunately, the exact etiology and pathogenesis of IPF remain elusive, with potential risk factors including genetic variations, long-term exposure to air pollution, smoking, certain viral infections, and gastroesophageal reflux disease [3]. Although anti-fibrotic drugs like pirfenidone and nintedanib, recommended by current guidelines, have displayed modest ability in slowing disease progression, halting or reversing the process of IPF remains a challenge [4]. Thus, the identification of novel drug targets capable of preventing IPF or delaying its progression assumes paramount significance.

**Fig. 1:**
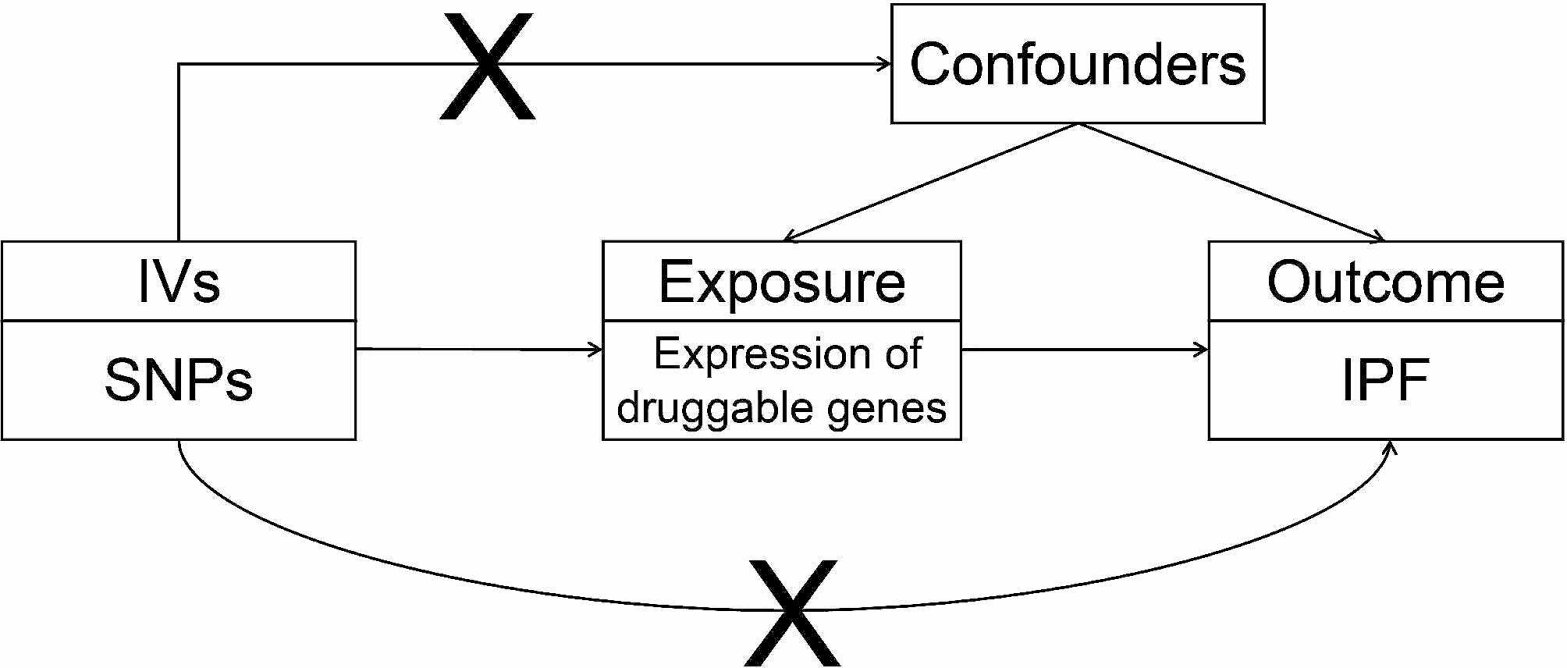
Overview of MR analysis. Choose cis-eQTL of druggable genes as instrumental variables (IVs) to investigate the causal relationship between the expression of druggable genes and IPF. The “X” between IVs and confounders indicates that the IVs are independent of any confounding factors The “X” between IVs and outcome indicates that the IVs only affect the outcome through the exposure rather than other potential pathways. IV, instrumental variable; SNP, single-nucleotide polymorphisms; IPF, Idiopathic pulmonary fibrosis

Mendelian randomization (MR) is an approach that employs genetic variants associated with specific exposures as instrumental variables to estimate causal relationships between the exposure of interest and the desired outcome (Fig. 1) [4]. Guided by the laws of gene segregation and independent assortment, alleles segregate and genes on non-homologous chromosomes recombine freely during gamete formation. Subsequently, the combination of parental gametes determines the presence or absence of certain genes, facilitating the random distribution of lifetime-long exposures. MR analysis has similar power to randomized controlled trial (RCT) with less bias and no reverse causality [5].

In drug target MR analysis, single nucleotide polymorphisms (SNPs) associated with gene expression levels, known as expression quantitative trait loci (eQTL), are employed as instrumental variables to examine the effects of druggable genes. Specifically, cis-eQTLs in the genomic regions proximal to the target gene are often selected due to their close relationship with gene expression. This methodology has garnered widespread application across various diseases, including Parkinson’s disease, aortic aneurysms, and even the COVID-19 [6–8].

Building upon this foundation, the present study aims to leverage the power of MR analysis to unearth potential drug targets for IPF from a pool of 4,479 druggable genes encoding drug targets or proteins related to drug targets through MR method, whether to prevent disease or delay the progression.

## Methods

### Study design

**Fig. 2:**
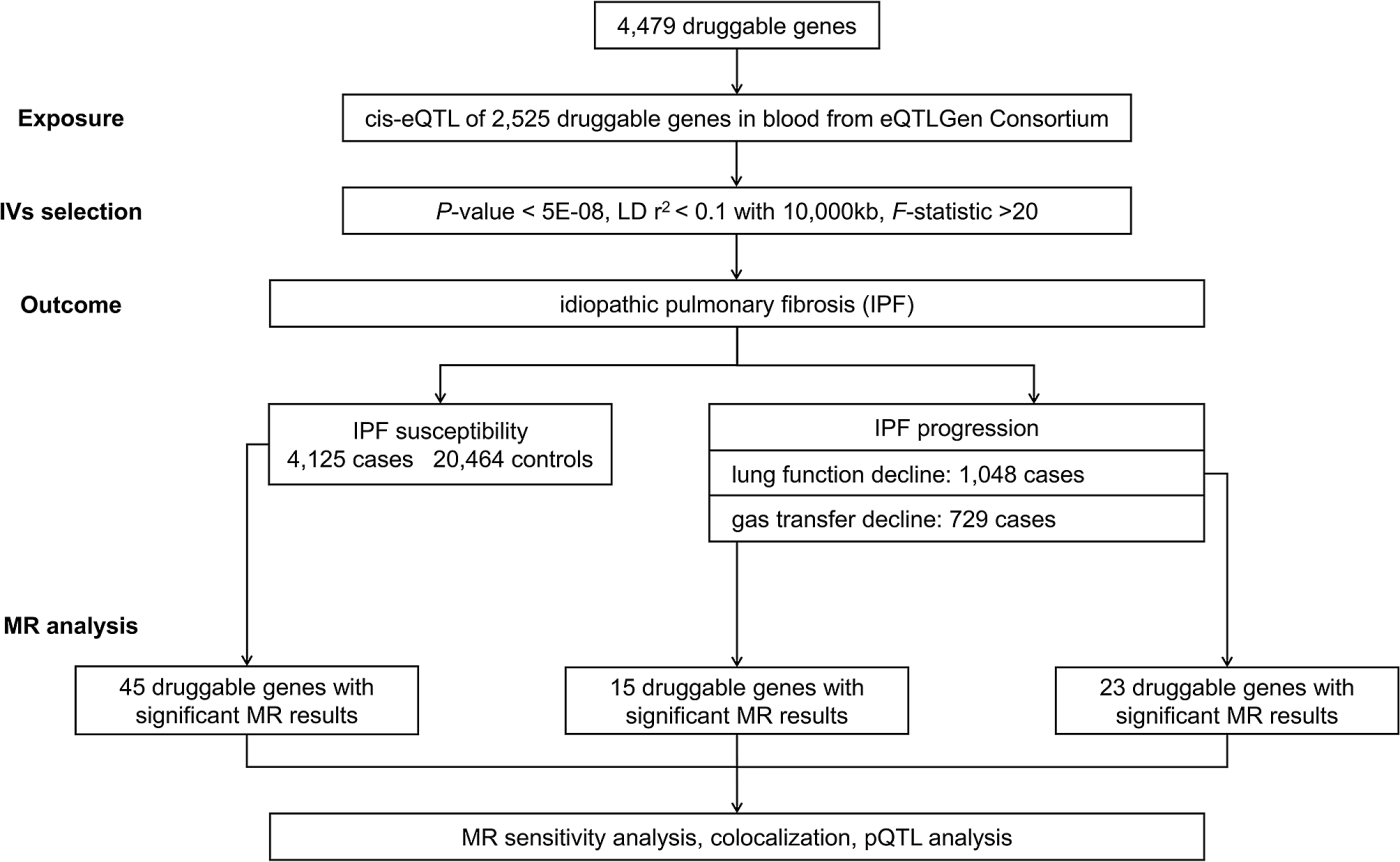
The flowchart of our study design. MR, mendelian randomization; eQTL, expression quantitative trait locus; pQTL, protein quantitative trait locus; FVC, forced vital capacity; DLco, diffusing capacity of the lungs for carbon monoxide

The flowchart visually describing the overall of the study is shown in Fig. 2. In short, we performed a two-sample MR analysis utilizing cis-eQTL of druggable genes in the blood as exposure and the genome wide association study (GWAS) of IPF as outcomes to investigate the causal relationship between the expression of druggable genes and susceptibility and progression of IPF. According to strict inclusion and exclusion criteria, appropriate SNPs were selected as instrumental variables (IVs). A series of sensitivity analyses was conducted to control the quality of MR analysis. For the druggable genes that exhibited significant MR results, we performed colocalization analysis to assess whether the same causal variant was shared by both the cis-eQTL and IPF. Additionally, we conducted protein quantitative trait locus (pQTL) analysis, which provided further validation of these druggable genes by examining the effect of the protein levels on the outcome.

### Exposure data

The druggable genes are defined as a set of genes encoding proteins with potential to be modulated by a drug-like small molecule based on sequence and structural similarity to the targets of existing drugs [9]. A total of 4,479 druggable genes were identified by Finan et al. including 1,427 genes encoding approved or clinical-phase drug targets, 682 genes encoding proteins that bind to known drug molecules or are similar to approved drug targets and 2,370 genes that were members of key druggable gene families or encoding proteins with distant similarity to approved drug targets [9]. This diverse collection of druggable genes offered a wide range of potential targets for investigation (Supplementary material: Table S2).

The cis-eQTL data in the blood for only 2,525 genes out of 4,479 druggable genes was obtained by searching in eQTLGen Consortium [10]. This consortium incorporates 37 datasets with a total of 31,684 individuals, predominantly of European ancestry. The eQTL data facilitates the identification of genetic variants associated with gene expression levels in blood samples, situated within a 1 Mb distance from the central location of each gene. The minor allele frequency (MAF) of every variant is greater than 0.01.

The pQTL data was available from the INTERVAL study encompassing 3,301 healthy participants of European descent [11]. In this study, a total of 1,927 pQTLs about 1,478 plasma proteins were identified. We selected the pQTL for druggable genes significantly colocalized with IPF outcomes to further investigate the relationship between levels of protein encoded by druggable genes and outcomes.

### Instrumental variables (IVs) selection

To ensure the reliability and accuracy of our results, it is crucial to satisfy three important assumptions in MR analysis: (1) The IVs are strongly associated with exposure; (2) The IVs are independent of any confounding factors; (3) There is no presence of horizontal pleiotropy, meaning that the IVs only affect the outcome through the exposure and not through any other potential pathways.

In line with these assumptions, a rigorous selection process was implemented for each druggable gene in our study. Firstly, we employed a stringent threshold and selected SNPs from the cis-eQTL data, ensuring that only those with *P*-values lower than the genome-wide significance threshold (5.0 × 10^− 8^) were considered. Next, in order to achieve a set of mutually independent SNPs, the SNPs for every druggable genes were clumped based on the 1,000 Genomes Project European population and the linkage disequilibrium (LD) threshold was set to r^2^ < 0.1 with a clumping window of 10,000kb [12]. Thirdly, incompatible SNPs between the exposures and outcomes (e.g., A/G vs. A/C) were excluded and positive strand alleles were inferred using allele frequencies for palindromes or the palindromic SNPs were excluded directly if there were no allele frequencies. Finally, the following formula was used to calculate the *F*-statistic [13].

$$F=\frac{N-k-1}{k}\times \frac{ { R}^{2}}{1-{R}^{2}}$$

The *F*-statistic serve as an essential metric in MR analysis, determining the strength of the IVs’ association with the exposure variable and aiding in the assessment of possible bias or weak instrument issues. In this formula, *R*^*2*^ is the proportion of variance explained by the IVs, *N* is the sample size, and *k* is the number of IVs. The SNPs with *F*-statistic less than 20 were excluded to avoid weak instrument bias [13].

### Outcome data

The GWAS statistics for IPF susceptibility and progression were obtained from the International IPF Genetics Consortium. For the GWAS of IPF susceptibility, a meta-analysis was conducted across five studies, comprising a total of 4,125 cases and 20,464 controls [14]. For the GWAS of IPF progression, two key measurements, namely forced vital capacity (FVC) and diffusing capacity of the lungs for carbon monoxide (DLco), were employed to identify variants that may contribute to a more rapid decline in lung capacity or gas transfer among IPF patients. There were 1,048 cases a total of 4,560 FVC measures and 729 cases with a total of 2,795 DLco measures [15].

### Mendelian randomization and colocalization

MR analysis was conducted using the R package “TwoSampleMR” (version 0.5.6). For the MR analysis, Wald ratio method was used when there was only one SNP as the IV. And inverse variance weighted (IVW), MR-Egger, weighted median, simple mode and weighted mode five methods were utilized if the IV contained two or more SNPs. Previous research has indicated that the IVW method is more conservative but robust compared to the other four methods [16]. Therefore, whether or not there is heterogeneity, the results were mainly based on the IVW method, supplemented by the others. To account for multiple testing, FDR (false discovery rate) corrections were applied to identify significant MR results.

Then the sensitivity analysis was performed by several methods. The potential heterogeneity of IVs was examined by Cochrane’s *Q* test [17]. If the *P*-value of Cochrane’s *Q* test was less than 0.05, it was indicative of heterogeneity. And MR-Egger regression was used to detect potential pleiotropy in the association between the exposures and outcomes [17]. If the *P*-value of MR-Egger regression intercept was less than 0.05, it suggested the presence of pleiotropy and rendered the MR analysis results unreliable.

For the druggable genes exhibiting significant MR results, colocalization analysis was conducted using R package “coloc” (version 5.1.0.1) [18]. The default prior probability was *P*1 = 1.0 × 10^− 4^, *P*2 = 1.0 × 10^− 4^, *P*12 = 1.0 × 10^− 5^, representing respectively a SNP is associated with the expression of the druggable genes, the outcome, or both. The posterior probabilities for the following 5 hypotheses were generated from colocation analysis: PPH0, no association with either expression of the druggable genes or outcome; PPH1, association with expression of the druggable genes, but not outcome; PPH2, association with outcome, but not expression of the druggable genes; PPH3, association with expression of the druggable genes and outcome, with different causal variants; PPH4, association with expression of the druggable genes and outcome, with a shared causal variant. PPH4 > 0.80 was considered strong evidence for colocalization and the genes colocalized with IPF were regarded as potential targets. The variant most closely associated with exposure (with the lowest *P*-value) was selected as the reference variant and variants ± 500 kb of the reference variant were included in colocalization analysis.

## Results

According to the selection criteria of IVs, a total of 4,0356 SNPs were used as IVs for 2,525 druggable genes. The *F*-statistic of IVs all exceeded 20, indicating no evidence of weak instrument bias. Details about the IVs are shown in Supplementary material: Table S3.

### Mendelian randomization

**Fig. 3:**
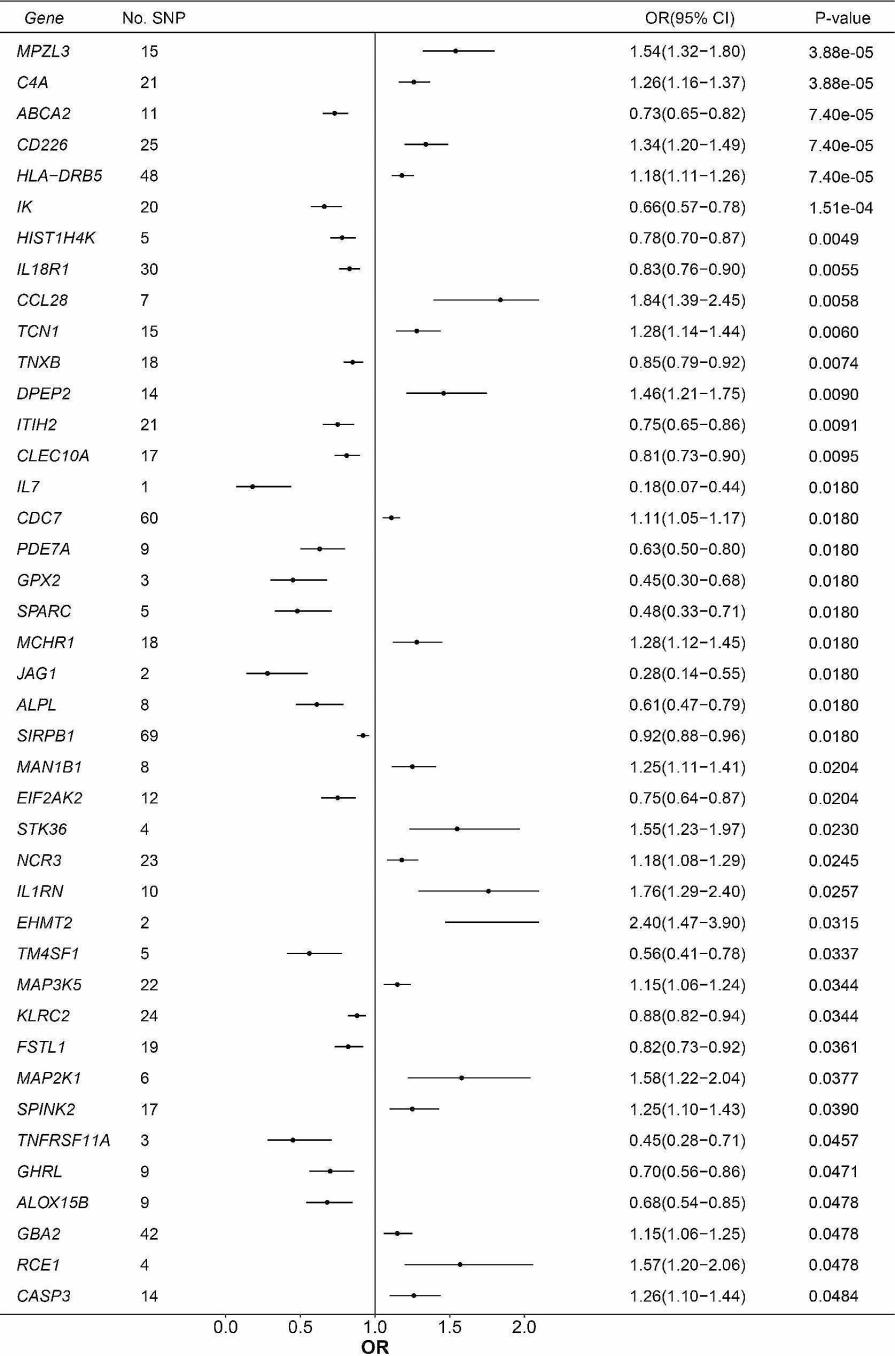
Significant MR results between the expression of druggable genes and IPF susceptibility after FDR correction

**Fig. 4:**
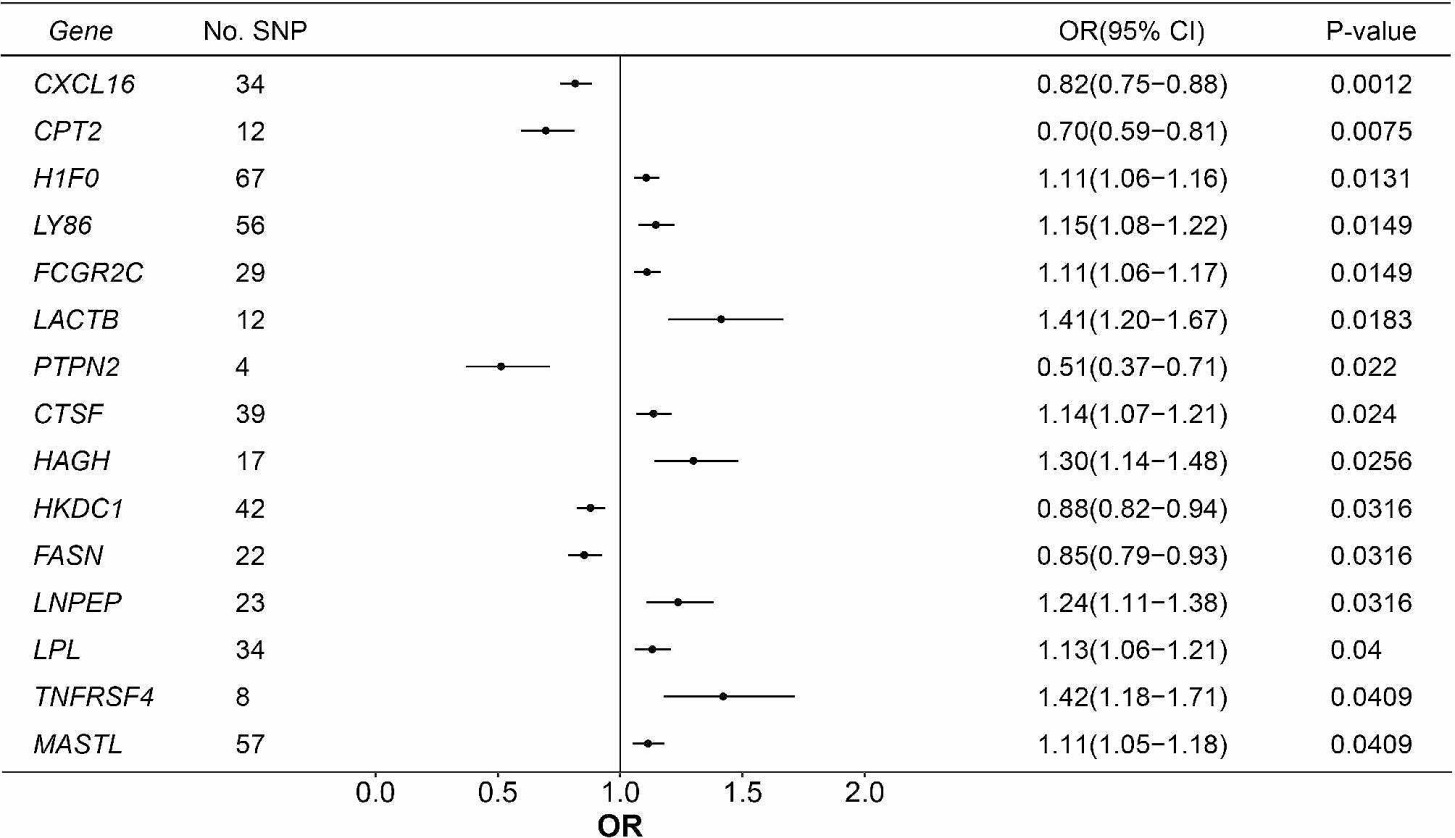
Significant MR results between the expression of druggable genes and DLco decline after FDR correction

Based on the IVW method, we found the expression of 45 druggable genes was significantly associated with IPF susceptibility at FDR < 0.05. The expression of 23 and 15 druggable genes was significantly associated with decreased FVC and DLco levels in IPF patients, respectively (Figs. 3 and 4).

The results of Cochran’s *Q* test showed no heterogeneity in IVs for significant genes (Supplementary material: Table S4-6). Furthermore, for some significant genes, pleiotropy was detected by MR-Egger regression methods and the corresponding results for these genes were considered unreliable (Supplementary material: Table S4-6).

### Colocalization

For the druggable genes with significant MR results, we conducted colocalization analysis to calculate probability of cis-eQTL and IPF outcomes sharing a causal variant. The results of colocalization analysis indicated IPF susceptibility and two significant genes (*IL-7* and *ABCA2*) were likely to share a causal variant, with a posterior probability of PP.H4 > 0.80% (*IL-7*: 84.00%, *ABCA2*: 81.50%). But there was no evidence of colocalization between IPF progression and the significant genes (Supplementary material: Table S7). Therefore, *IL-7* and *ABCA2* were identified as potential drug targets for reducing IPF risk based on MR and colocalization analyses.

### pQTL analysis for *IL-7*

To verify the effect of druggable gene expression on IPF susceptibility, we further investigated plasma protein levels using pQTL data. The pQTL data for *IL-7* was obtained from the INTERVAL study. Unfortunately, we could not find any pQTL data for *ABCA2*.

We filtered out the SNPs with *P*-values less than the genome-wide significance threshold and clumped with r^2^ < 0.001 and clumping window of 10,000 kb. Only one SNP (rs72673751) was screened as IV representing IL-7 protein level for pQTL analysis (Supplementary material: Table S8). To ensure the validity of result, we searched on PhenoScanner website to exclude the existence of pleiotropy which could affect outcome through potentially other pathways.

The results of the pQTL analysis demonstrated that high levels of IL-7 in plasma are associated with a reduced risk of IPF (OR = 0.67, 95%CI: 0.47–0.97, *P* = 0.035), which is consistent with the findings of eQTL analysis (Supplementary material: Table S9).

## Discussion

In order to identify potential drug targets for IPF, we conducted a large-scale MR analysis to evaluate the role of 2,429 druggable gene expression in IPF susceptibility and progression. After a series of sensitivity analyzes and further analyses, including Cochrane’s *Q* test, MR-Egger regression, colocalization analysis, pQTL analysis, we have discovered that *IL-7* holds the most promising potential as a therapeutic target for IPF susceptibility. However, it is important to note that the therapeutic effect of *IL-7* was not replicated in the IPF progression cohort.

Although the pathogenesis of IPF has not been fully elucidated, there is sufficient evidence that transforming growth factor–β (TGF-β) plays a key role. Overexpressed TGF-β induces epithelial-mesenchymal transition (EMT) and promotes abnormal deposition of extracellular matrix (ECM), leading to pulmonary fibrosis [19]. There have been some studies exploring how IL-7 affects TGF-β to reduce the risk of IPF. Huang et al. [20] demonstrated that IL-7 can not only down-regulate the synthesis of TGF-β in lung fibroblasts but also block TGF-β signaling through the intact JAK1/STAT1 pathway to reduce collagen synthesis. In addition, further studies found that IL-7 also inhibited PKC-δ activity to reduce TGF-β-induced expression of collagen genes *COL1A1* and *COL3A1* [21]. They also found that IL-7 was able to alleviate bleomycin-induced pulmonary fibrosis in vivo [20]. In an observational study using direct hemoperfusion with a polymyxin B-immobilized fiber column (PMX-DHP) for acute exacerbations of IPF, plasma IL-7 level was significantly higher in survivors compared with non-survivors on day 30 after treatment, which may indicate IL-7 has potential anti-fibrotic effects [22]. These previous studies suggest that IL-7 has therapeutic potential for IPF. Different from the perspective of the above studies, our study proved this genetically through MR analysis.

Some MR analyzes about IPF have been published, including lung cancer, gastroesophageal reflux disease, allergic rhinitis, but our study is the first to apply drug target MR analysis using eQTL to IPF. One of the strengths of our study lies in the size and diversity of the GWAS data used. To the best of our knowledge, these GWAS data are currently the largest available for IPF research. Furthermore, we ensured that there was no overlap between the population samples used in different GWAS, which adds to the reliability and validity of our findings. We implemented strict screening criteria during the IVs selection process. By following these stringent procedures and ensuring the fulfillment of key assumptions, we aimed to minimize the risk of bias and obtain reliable results in our MR analysis. These rigorous steps were essential in upholding the validity and integrity of our findings, thereby bolstering the overall robustness of our study. Colocalization analysis showed that IL-7 and IPF are likely to share the same causal variant, which strengthens the causal relationship. Of course, this result may be caused by pleiotropy [23, 24]. But our study using cis-eQTL variants is supported by a clear and unidirectional biological principle (the central dogma) with less likelihood of other pathways, reducing potential horizontal pleiotropy [15]. In addition to IL-7, our study also identified other targets. Although they were not supported by colocalization analysis, their potential value cannot be completely denied, still providing broad possibilities for the development of IPF drugs.

There are several limitations in our study. Drug target MR only simulates the lifetime low-dose exposure of drugs under ideal conditions, and the actual situation will be more complicated due to the interference of other factors, so it cannot completely replace clinical trials and the actual efficacy of drugs is uncertain. Therefore, clinical trials remain necessary, and our study provides valuable insight and direction for the development of new drugs for IPF. Secondly, MR can only evaluate the impact of single druggable gene expression on outcome separately. However, many drugs exert their effects through the superposition of multiple targets. Thirdly, this study only included eQTL in blood, because we did not obtain appropriate eQTL data in the lung tissue. In case of unavailability of eQTL data in the lung tissue, biomarkers from the lungs will be released into the blood in the context of disease and blood serves as a valuable proxy tissue that offers a systemic perspective on disease processes. Blood carries molecular signals and cellular components from various organs and tissues, to a certain extent reflecting the dynamic interplay of systemic processes. The choice of blood has its limitations, including the dilution effect of systemic circulation and the potential masking of tissue-specific signals. Some molecular signals of the disease may not be fully revealed in blood eQTLs. Fortunately, some experiments [20, 21] have demonstrated the anti-fibrotic effect of IL-7 in lung tissue, which makes up for this deficiency in our study. Furthermore, it is important to note that some studies have pointed out that the inhibition of TGF-β will show a variety of side effects, due to its wide range of effects [19]. And high levels of IL-7 are associated with autoimmune diseases such as rheumatoid arthritis [25], whether boosting IL-7 would have similar side effects as inhibiting TGF-β or more is not known, which may limit the application of IL-7 boosting strategy to IPF patients. Finally, the participants in the GWAS used were almost exclusively of European ancestry. This restriction may limit the generalizability of our results to other populations.

## Conclusions

Drug target MR opens a new avenue for identifying potential drug targets utilizing druggable genetic data and disease GWAS data. In conclusion, through the drug target MR analysis based on the druggable genes, we have found that *IL-7* holds promise as a potential target to reduce the risk of IPF in high-risk population. However, it is imperative to conduct further research to validate the effect of *IL-7* in preventing IPF.

### Electronic supplementary material

Below is the link to the electronic supplementary material.

Supplementary Material 1

## Data Availability

All data used in this study are publicly available and listed in Table S1. The cis-eQTL data were obtained from the eQTLGen Consortium (https://www.eqtlgen.org/cis-eqtls.html). The pQTL data was available from the INTERVAL study (https://gwas.mrcieu.ac.uk/datasets/prot-a-1543/). The GWAS statistics for IPF susceptibility and progression were obtained from the International IPF Genetics Consortium (https://github.com/genomicsITER/PFgenetics).

## Abbreviations

IPF: Idiopathic pulmonary fibrosis
eQTL: Expression quantitative trait locus
pQTL: Protein quantitative trait locus
GWAS: Genome wide association study
FVC: Forced vital capacity
DLco: Diffusing capacity of the lungs for carbon monoxide
MR: Mendelian randomization
SNP: Single nucleotide polymorphism
IV: Instrumental variable
IVW: Inverse variance weighted
FDR: False discovery rate
TGF-β: Transforming growth factor–β
